# Long-term extension data do not robustly support clinical disease course modification with donanemab

**DOI:** 10.1016/j.tjpad.2026.100511

**Published:** 2026-02-20

**Authors:** Jemma Hazan, Kathy Y. Liu, Robert Howard

**Affiliations:** a Division of Psychiatry, University College London, London, UK; b North London NHS Foundation Trust, London, UK

**Keywords:** Alzheimer's disease, Dementia, Amyloid, Clinical trial

Zimmer and colleagues report the results of a 78-week participant- and investigator-blinded long-term extension (LTE) of the 76-week TRAILBLAZER-ALZ 2 trial of donanemab [1]. Participants initially randomised to placebo were given an opportunity to receive blinded donanemab treatment on entering the LTE period, allowing comparisons to be made between “early-start” and “delayed-start” treatment groups at the end of the LTE. While the authors claim that donanemab treatment “slowed disease progression among early-start participants” and “successfully modifies the course of early symptomatic AD”, we believe these claims are not adequately supported by the study design and analysis and conclusions should have been worded more cautiously in light of these limitations.

Efficacy in the LTE period was evaluated independently for the early- and late-start groups against an external control population selected from ADNI and very few direct quantitative comparisons between early- and delayed-start participants are presented. They accept that their use of an external control cohort has limitations because of differences in study conduct and assessments, time periods and geographic regions of data collection and other potential unmeasured confounding factors which would include the attention, expectancy and placebo effects associated with participating in a clinical trial. They also acknowledged that delayed-start participants entered the long-term extension with more advanced disease than early-start participants, which may bias efficacy comparisons in favour of the early-start group.

The authors stated that the 27 % reduced risk of progressing to the next stage of disease as assessed by the CDR-G score in early- versus delayed-start participants “provided the best evidence of clinical meaningfulness from the available LTE data”. However, this interpretation relies on implicit within-individual causal comparisons that are not supported by the study’s analytical framework [2].

In Fig. 2, the authors present clinical efficacy, measured by change from baseline in CDR-SB, in three separate graphs for early-start, delayed-start and an early-start group who met specific treatment course completion criteria. Although early- and delayed-start participant data are not directly compared within the same graph, visual inspection of Fig. 2(a) and 2(b) indicates that by the end of the LTE at 36 months, both groups show a similar degree of worsening from baseline, approximately 4 CDR-SB points. Reported time saving based on the CDR-SB of only 1.3 months gives further quantitative support for this apparent convergence. Since early-start participants had shown a 0.6 CDR-SB points smaller worsening than the delayed-start group in the TRAILBLAZER-ALZ 2 primary outcome at 18 months, convergence at 36 months implies a relative acceleration of decline in the early-start participants, or equivalently, a “catch up” of the delayed-start group during the LTE.

Delayed start trial designs offer convincing demonstration of disease course modification in trials of dementia treatment when a group with delayed access to treatment does not catch up with the clinical benefit seen in the group who received early treatment. This is important for a drug like donanemab because persistence of a difference between groups would reflect an effect of treatment on underlying disease pathology and positive modification of clinical course, rather than a reversable symptomatic effect as seen with cholinesterase inhibitors and memantine. From what we can tell from both the graphical presentation of the primary outcome CDR-SB and the tiny time saving estimation reported from the LTE, there was no advantage to early-start treatment or to treating earlier in the clinical course of Alzheimer’s disease.

Apparent reluctance to present or discuss within-trial comparisons between early- and delayed-start participants is therefore surprising, particularly since these are the exploratory analyses that can best inform arguments about whether donanemab is able to modify the clinical course of Alzheimer’s disease so that hopes of time savings and longer-term benefits with treatment can be most fairly evaluated. We trust that the authors and their Sponsor will move to promptly publish these analyses and data.

## Funding

RH and KL are supported by 10.13039/501100000272National Institute for Health Research (NIHR) University College London Hospitals Biomedical Research Centre. JH is supported by the UCL ADAPT study (Blood Biomarker Challenge) and an Alzheimer’s Research UK Clinical Research Training Fellowship (CRTF2023B-003).

## Declaration of the use of generative AI and AI-assisted technologies in scientific writing and in figures, images and artwork

No generative AI or AI-assisted technologies were used

## CRediT authorship contribution statement

**Jemma Hazan:** Conceptualization, Methodology, Writing – original draft, Writing – review & editing. **Kathy Y. Liu:** Conceptualization, Writing – original draft, Writing – review & editing. **Robert Howard:** Supervision, Writing – original draft, Writing – review & editing.

## Declaration of competing interest

The authors declare the following financial interests/personal relationships which may be considered as potential competing interests.Jemma Hazan reports financial support was provided by Alzheimer’s Research UK. Kathy Y. Liu reports financial support was provided by NIHR University College London Hospitals Biomedical Research Centre. Robert Howard reports financial support was provided by NIHR University College London Hospitals Biomedical Research Centre. Robert Howard reports a relationship with Synaptogenix that includes: board membership. If there are other authors, they declare that they have no known competing financial interests or personal relationships that could have appeared to influence the work reported in this paper.
